# A first-in-human study of [^68^Ga]Ga-CDI: a positron emitting radiopharmaceutical for imaging tumour cell death

**DOI:** 10.1007/s00259-022-05880-z

**Published:** 2022-07-02

**Authors:** Ivan Ho Shon, Thomas Hennessy, Jennifer Guille, Michael P. Gotsbacher, Angelina J. Lay, Bruce McBride, Rachel Codd, Philip J. Hogg

**Affiliations:** 1 Department of Nuclear Medicine and PET, Prince of Wales Hospital, Sydney, Australia; 2 The Centenary Institute, University of Sydney, Sydney, Australia; 3 Prince of Wales Clinical School, University of New South Wales, Sydney, Australia; 4 School of Medical Sciences, University of Sydney, Sydney, Australia

**Keywords:** Cell death, Apoptosis, Necrosis, Positron emission tomography, Positron emission tomography computed tomography, Gallium-68

## Abstract

**Purpose:**

This study assesses human biodistribution, radiation dosimetry, safety and tumour uptake of cell death indicator labelled with ^68^Ga ([^68^Ga]Ga-CDI), a novel radiopharmaceutical that can image multiple forms of cell death.

**Methods:**

Five participants with at least one extracranial site of solid malignancy > 2 cm and no active cancer treatment in the 8 weeks prior to the study were enrolled. Participants were administered 205 ± 4.1 MBq (range, 200–211 MBq) of [^68^Ga]Ga-CDI and 8 serial PET scans acquired: the first commencing immediately and the last 3 h later. Participants were monitored for clinical, laboratory and electrocardiographic side effects and adverse events. Urine and blood radioactivity was measured. Spherical volumes of interest were drawn over tumour, blood pool and organs to determine biodistribution and calculate dosimetry. In one participant, tumour specimens were analysed for cell death using terminal deoxynucleotidyl transferase dUTP nick end labelling (TUNEL) staining.

**Results:**

[^68^Ga]Ga-CDI is safe and well-tolerated with no side effects or adverse events. [^68^Ga]Ga-CDI is renally excreted, demonstrates low levels of physiologic uptake in the other organs and has excellent imaging characteristics. The mean effective dose was 2.17E − 02 ± 4.61E − 03 mSv/MBq. It images constitutive tumour cell death and correlates with tumour cell death on histology.

**Conclusion:**

[^68^Ga]Ga-CDI is a novel cell death imaging radiopharmaceutical that is safe, has low radiation dosimetry and excellent biodistribution and imaging characteristics. It has potential advantages over previously investigated radiopharmaceuticals for imaging of cell death and has progressed to a proof-of-concept trial.

**Trial registration:**

ACTRN12621000641897 (28/5/2021, retrospectively registered)

**Supplementary Information:**

The online version contains supplementary material available at 10.1007/s00259-022-05880-z.

## Introduction

Assessment of response in oncology ultimately aims to measure tumour cell death following treatment. Cell death may be directly assessed in vitro and ex vivo by light microscopy, histochemical (e.g. annexin V staining, terminal deoxynucleotidyl transferase dUTP nick end labelling [TUNEL]) and immunohistochemical (e.g. anti-caspase 3 antibodies) techniques. However, these require invasive and potentially morbid sampling procedures which may be difficult to perform repeatedly for serial dynamic assessment and such samples are small and may not be representative. Presently, imaging response assessment in oncology relies on indirect measurements of tumour cell death, most often assessing changes in tumour size using x-ray computed tomography (CT) assessed by Response Evaluation Criteria in Solid Tumours (RECIST) [1] or changes in metabolic activity using positron emission tomography (PET) with 2-fluoro-2-deoxyglucose (FDG) [2]. These modalities are imperfect surrogates for tumour cell death.

Imaging of tumour cell death has the potential to overcome all the limitations of current clinical imaging for assessment of treatment efficacy. However, despite active investigation for over 20 years, it remains an unrealised opportunity. The most extensively investigated approach utilised the targeting of phosphatidylserine, which is externalised during apoptosis, with radiolabelled annexin V. Annexin V labelled with ^99m^Tc was demonstrated to bind to and image apoptotic tumour cells [3] and increased tumour uptake of ^99m^Tc-labelled annexin V has been observed in response to chemotherapy [4] and radiotherapy [5]. However ^99m^Tc-labelled annexin V has high levels of physiologic uptake particularly in the kidneys and liver [6] and has not progressed into clinical practice [7]. Radiolabelled isatin-based inhibitors of activated caspase 3/7 have also been investigated for imaging of cell death. One of these, [^18^F]F-ICMT-11, underwent a first-in-human study which demonstrated high levels of hepatobiliary excretion that makes imaging of the abdomen and pelvis sub-optimal [8]. A proof-of-concept study was unsuccessful in imaging an increase in tumour cell death in response to chemotherapy in patients with breast and lung cancer [9].

4-(*N*-(*S*-glutathionylacetyl)amino)phenylarsonous acid (GSAO) is a tripeptide trivalent arsenical that when conjugated at the γ-glutamyl residue with fluorescent or radionuclide reporter probes is unable to enter viable cells. However, GSAO conjugates enter dying cells via organic anion transporters whether death is via apoptotic or non-apoptotic pathways [10]. Once in the cytosol, the As(III) atom of GSAO cross-links cysteines 597 and 598 of heat shock 90 proteins (hsp90) forming a covalent complex that prevents washout of the label from the cell [10]. Hsp90 is a highly abundant and stable target in dying/dead cells. GSAO conjugate labelling of dying/dead cells is rapid, specific and saturable, with intensity of labelling of dying/dead cells at least 1000-fold higher than that of viable cells [10]. In a murine colorectal carcinoma model following treatment with doxorubicin, tumour cells that stained with GSAO-fluorophore conjugates also stained for activated caspase 3 [10]. In an orthotopic murine mammary carcinoma model, GSAO fluorophore conjugates detected cyclophosphamide-induced tumour cell death that correlated with TUNEL-positive cells in sectioned tumours [11].

Initial in vivo studies of radionuclide conjugates of GSAO demonstrated that ^111^In conjugated to GSAO with diethylene triamine pentaacetic acid (DTPA) non-invasively imaged tumour cell death concordant with ^99m^Tc-labelled annexin V with relatively lower levels of uptake in normal tissues and organs except for the kidneys [10]. GSAO was conjugated with ^67^Ga using 1,4,7,10-tetraazacyclododecane-1,4,7,10-tetraacetic acid (DOTA) and this demonstrated higher tumour uptake and lower uptake in normal tissues and organs compared to [^111^In]In DTPA GSAO [12]. Subsequently, a robust, convenient and broadly applicable method for labelling GSAO with ^68^Ga to enable short interval serial PET imaging was developed using the bifunctional chelator 2-(4,7-bis(carboxymethyl)-1,4,7-triazonan-1-yl)pentanedioic acid (NODAGA) [13], hereafter referred to as [^68^Ga]Ga-Cell Death Indicator ([^68^Ga]Ga-CDI). In vivo studies demonstrated favourable biodistribution and imaging characteristics and predicted dosimetry similar to clinical ^68^Ga radiopharmaceuticals suitable for human use [14]. This study aimed to assess the first-in-human biodistribution, radiation dosimetry, safety and tumour uptake of [^68^Ga]Ga-CDI.

## Material and methods

### Participants

This open label, single arm interventional study recruited participants with histologically or cytologically confirmed solid malignancy with at least one measurable lesion > 2 cm. The study was approved by the South Eastern Sydney Local Health District Human Research Ethics committee (2019/ETH04821) and all participants signed an informed consent form. The study is registered with the Australian New Zealand Clinical Trials Registry (ACTRN12621000641897) and is registered with the Therapeutics Goods Administration of the Department of Health, Australian Government under the Clinical Trials Notification scheme (CT-2018-CTN-00827–1). Within 28 days of undergoing the study, participants were screened to ensure they met inclusion and exclusion criteria and written consent obtained. The full inclusion and exclusion criteria are shown in Table 1. Although not an inclusion criteria for participation in this study, all patients also underwent an FDG PET CT between 3 and 11 days prior to CDI PET CT as part of standard of care.

**Table 1:** Inclusion and exclusion criteria

Inclusion criteria	Exclusion criteria
Able to understand and willing to sign the written informed consent Male or female patients ≥ 18 years of age Histologically or cytologically confirmed solid malignancy with at least one measurable lesion > 2 cm Within 28 days of commencement: Adequate liver function (bilirubin ≤ 1.5 upper limit normal (ULN), ALT and AST ≤ 4 ULN) Adequate renal function (eGFR > 50 mL/min/1.73 m^2^) Adequate bone marrow function (absolute neutrophil count ≥ 1.5 × 10^9^/L, haemoglobin level ≥ 9.0 g/dL and platelets ≥ 100 × 10^9^/L) Serum potassium ≥ 3.0 mmol/L and magnesium ≥ 0.6 mmol/L	Cancer treatment within the previous 6 weeks Primary or isolated metastatic CNS malignancy Active uncontrolled infection Congestive heart failure or prior NYHA class III–IV cardiac disease Uncontrolled hypertension (systolic BP > 180 mmHg or diastolic BP > 100 mmHg) Evidence of recent heart disease (myocardial infarction in the past 2 months by ECG, arrhythmias associated with QTc prolongation or evidence of ischemia) QTc > 480 ms Medications that prolong QTc Pregnancy Breast feeding

### [^68^Ga]Ga-CDI PET CT protocol and monitoring

[^68^Ga]Ga-CDI was prepared as previously described. In summary, CDI was first prepared by dissolving GSAO in 0.1 N sodium bicarbonate. 2,2′-(7-(1-carboxy-4-((2,5-dioxopyrrolidin-1-yl)oxy)-4-oxobutyl)-1,4,7-triazonane-1,4-diyl)diacetic acid (NODAGA-NHS, Chematech) was dissolved in anhydrous dimethylformamide and added to the reaction mixture dropwise over 1 h. The reaction mixture was stirred for 4 h, acidified by the addition of 1 N hydrochloric acid, shock-frozen in liquid nitrogen and freeze-dried. The residue was redissolved in de-aerated water and purified by high performance liquid chromatography (HPLC). Purity was confirmed by liquid chromatography–mass spectroscopy (LC–MS) and frozen in 100 µL aliquots (54 µg).

CDI was radiolabelled by eluting [^68^ Ga]Cl_3_ from the Ge/Ga generator (Eckert and Ziegler-IGG-100) onto a cation exchange cartridge (Agilent Bond Elut SCX). ^68^ Ga (III) was then eluted from the cation exchange cartridge using a mixture of hydrochloric acid (HCl) and sodium chloride (NaCl) (12.5 µL of 5.5 N HCl and 500 µL of 5 N NaCl) into a reaction vial containing 54 µg of CDI, ascorbic acid (4.4 mg in 100 µl), sodium acetate buffer (250 µL, 1.5 N, pH 4.5) and 3.5 mL water (Ultrapur). The reaction vial was incubated at room temperature for 10 min. Phosphate buffer (3 mL, 384 mM, pH 7.9) was added and the mixture filtered through a 0.22-µm filter (Millipore Millex-GV 33 mm). Quality control was performed with HPLC and thin layer chromatography (TLC) [13].

Participants were encouraged to be well orally hydrated prior to [^68^Ga]Ga-CDI administration; otherwise, there was no preparation required. Two intravenous cannulas were placed (one for administration of [^68^Ga]Ga-CDI and the other for blood sampling during the study). Immediately prior to [^68^Ga]Ga-CDI administration, clinical assessment of participants was performed, vital signs (blood pressure, heart rate, respiratory rate, arterial oxygen saturation and temperature) measured and a 12-lead electrocardiogram (ECG) obtained and QTc calculated according to Bazett’s formula to confirm eligibility. [^68^Ga]Ga-CDI was administered as an intravenous bolus. The mean and standard deviation of the administered mass of [^68^Ga]Ga-CDI was 40.1 ± 5.8 µg (range, 32.8–48.8 µg). The mean and standard deviation of administered activity was 205 ± 4.1 MBq (range 200–211 MBq) of [^68^Ga]Ga-CDI.

Eight serial PET scans were then performed from skull vertex to the proximal femora. The first PET scan commenced immediately following administration of [^68^Ga]Ga-CDI, with subsequent scans commencing at approximately 7, 17, 29, 47, 64, 120 and 180 min after administration (+ / − 5 min). All scans were performed on a Phillips ingenuity TF 128 PET CT scanner (Philips Medical Systems, Cleveland) with acquisition times increasing for each subsequent scan ranging from 30 (immediately after administration) to 240 s/bed (at 180 min after administration) to compensate for excretion and decay. All PET scans were reconstructed using CT attenuation correction with a time of flight, list-mode, blob-based, ordered subsets maximum likelihood expectation maximisation algorithm (BLOB-OS-TF) (3 iterations, 3 subsets, kernel width = 18.1 cm, relaxation parameter = 1, reconstructed voxel size 4 × 4 × 4 mm).

Immediately prior to the sixth PET scan commencing at approximately 47 min post administration, a low-dose, non-contrast CT of the same region was performed for attenuation correction and localisation (of the first six scans during which time the patient had not moved) (44–120 mAs, 120–140 kV adjusted for body weight and body mass index, iDose 3 iterative reconstruction, reconstructed voxel size 1.17 × 1.17 × 3 mm). Ultra-low dose non-contrast CT (25 mAs, 100 kV, filtered back projection reconstruction, reconstructed voxel size 1.17 × 1.17 × 3 mm) was performed for attenuation correction immediately prior to the 120-min and 180-min PET scans (previous work validated the quantitative accuracy of these ultra-low-dose CT for attenuation correction) [15].

During and immediately following completion of the scanning protocol, participants were clinically assessed for adverse reactions and vital signs measured. Blood was collected for venous radioactivity measurements at 1, 2 and 3 h following [^68^Ga]Ga-CDI administration. Participants were asked to void between 1.5 to 2 h and 2.75 to 3 h after [^68^Ga]Ga-CDI administration, urine collected, the volume measured and an aliquot counted in a cross calibrated gamma counter to determine activity concentration. Following completion of the scanning protocol, ECG was recorded, and blood was collected for biochemical and haematological assessment.

### Post [^68^Ga]Ga-CDI PET CT monitoring

On the day following the [^68^Ga]Ga-CDI PET CT, participants were clinically assessed for adverse reactions and vital signs, ECG was obtained, and blood was collected for biochemical and haematological assessment. Clinical follow-up was also performed 7 days later for assessment of adverse reactions.

### Analysis of [^68^Ga]Ga-CDI PET CT scans

For analysis of uptake and biodistribution in normal tissues and organs, spherical volumes of interest (VOIs) were drawn on the Digital Imaging and Communications in Medicine (DICOM) [^68^Ga]Ga-CDI-PET emission data on a workstation (IntelliSpace Portal, V5.0.2.60000, Philips Medical System, Best, The Netherlands) by an experienced nuclear medicine physician over clearly tumour-free regions of organs and the left ventricular and aortic blood pool (the average of which was used for blood pool activity). The imaging-derived blood pool activity was used for biodistribution and dosimetry calculations (the blood pool activity measured from the venous blood samples collected correlated closely with the imaging-derived blood pool activity but was not used for calculation of biodistribution and dosimetry). VOI diameter varied depending on organ size but where possible was at least 10 mm. Spherical VOIs were also drawn over all tumour deposits greater than 10 mm in diameter. These VOIs were replicated across all time points and the mean, maximum and standard deviation of the activity concentration (Bq/mL) of all VOIs at each time point was recorded. Activity concentration was used to calculate the % injected activity (%IA) for organs and tissues based on the masses of source organs for standard phantom male and female models [16]. Mean, maximum and standard deviation of the SUV for each VOI was also a calculated from the activity concentration using the slope intercept stored with the DICOM CDI PET emission data.

### Radiation dosimetry 

Activity concentrations derived above were entered into OLINDA/EXM 1.1 and bi-exponential curves fitted using the curve fitting tools within OLINDA/EXM 1.1 [17]. A urinary bladder filling and voiding model were derived. Organ and total body effective dose was calculated using ICRP publication 103 weighting factors [18].

### Histological correlation

In one participant, who proceeded to surgical excision of the tumour deposits 1 day after the [^68^Ga]Ga-CDI PET study, histological correlation of the tumour deposits was undertaken using haematoxylin and eosin staining and TUNEL staining to specifically identify dead and dying cells. Representative samples of the formalin fixed surgical specimens (following completion of all clinical analysis and excess to that required for archival purposes) from the right axillary and right cervical nodal masses were selected and provided by the reporting clinical pathologist. The tissues were fixed, rehydrated with ethanol and xylene and embedded in paraffin. A series of 4 µm thick sections were cut and stained with haematoxylin and eosin. Apoptotic cells were stained using the TUNEL assay kit HRP-DAB (Abcam, Cat#206,386, Cambridge, MA, USA) according to manufacturer’s protocol. Briefly, 4-µm sections of paraffin embedded tumour were deparaffinised in xylene and rehydrated in decreasing concentrations of ethanol. Tumour sections were permeabilized with Proteinase K for 20 min at room temperature. The endogenous peroxidase activity was quenched with 3% H_2_O_2_ for 5 min. Apoptotic cells were labelled with biotinylated Terminal deoxynucleotidyl Transferase (TdT) at 37 °C in a humidified chamber for 2 h followed by a 30-min incubation with streptavidin-HRP conjugate. HRP-positive cells were developed using diaminobenzidine (DAB) and specimens were counterstained with methyl green (Sigma, St Louis, MO, USA). The entire tumour section was imaged using PowerMosaic scanning at 10 × magnification on a Leica DM6000D microscope.

## Results

### Participants

Five participants were recruited to this study, three females and two males, ranging in age from 52 to 81 years (Table 2).

**Table 2:** Participant characteristics

Participant	Age	Gender	Diagnosis	Site(s) of disease	Previous treatment(s)	Interval since last treatment
1	66	M	Squamous cell carcinoma of oesophagus	Upper oesophagus	New diagnosis	Not applicable
2	73	F	High grade serous carcinoma of the ovary	Peritoneum	Surgery, chemotherapy	12 weeks
3	66	F	Squamous cell carcinoma (cutaneous)	Lymph nodes (right upper anterior cervical triangle and right axilla)	New diagnosis	Not applicable
4	81	F	Grade II invasive ductal breast carcinoma, oestrogen receptor–positive, HER-2-negative	Right breast and right axillary lymph node	New diagnosis	Not applicable
5	52	M	Adenocarcinoma (colon)	Liver and retroperitoneal lymph nodes	Multiple surgeries, chemotherapy	11 months

### Safety and tolerability

[^68^Ga]Ga-CDI was well-tolerated. There were no adverse or clinically detectable pharmacologic effects in any of the 5 participants. No significant changes in vital signs or the results of laboratory studies or electrocardiograms were observed.

### Biodistribution and imaging

**Fig. 1:**
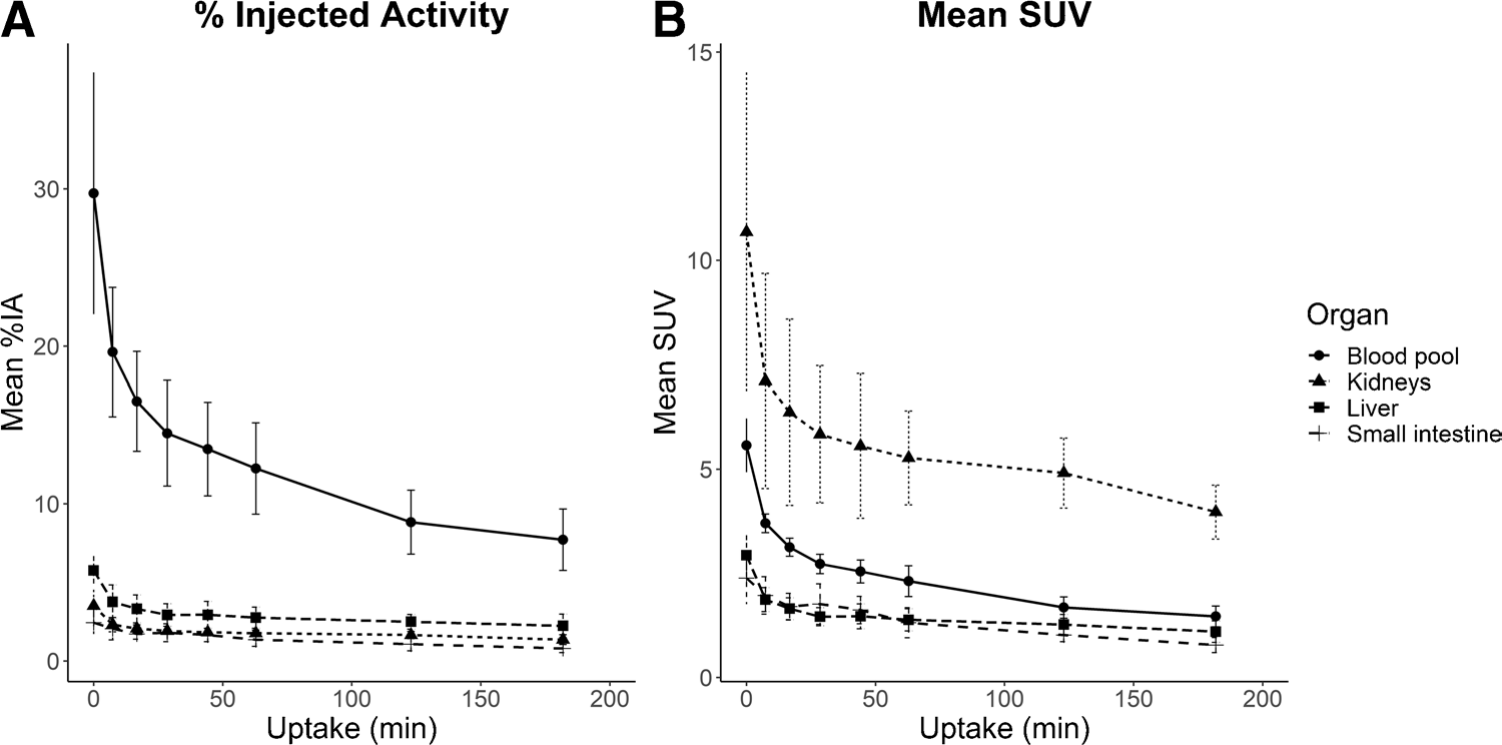
Mean %IA (**A**) and SUV (**B**) for selected tissues/organs

Following [^68^Ga]Ga-CDI administration, there is rapid distribution in the blood pool, with rapid renal uptake and excretion. Of the organs and tissues, the greatest amount of activity is within the blood pool with on average 12.2%, 8.8% and 7.7% IA remaining in the blood pool at 1, 2 and 3 h, respectively. The highest concentration of activity is within the kidneys with an average SUV of 5.3, 4.9 and 4.0 at 1, 2 and 3 h post injection, respectively (Fig. 1). The remaining organs demonstrated lower activity concentrations and there was a progressive decline in activity in all organs over time. By 90 min, 39 ± 18% of total activity has been excreted in urine.

**Fig. 2:**
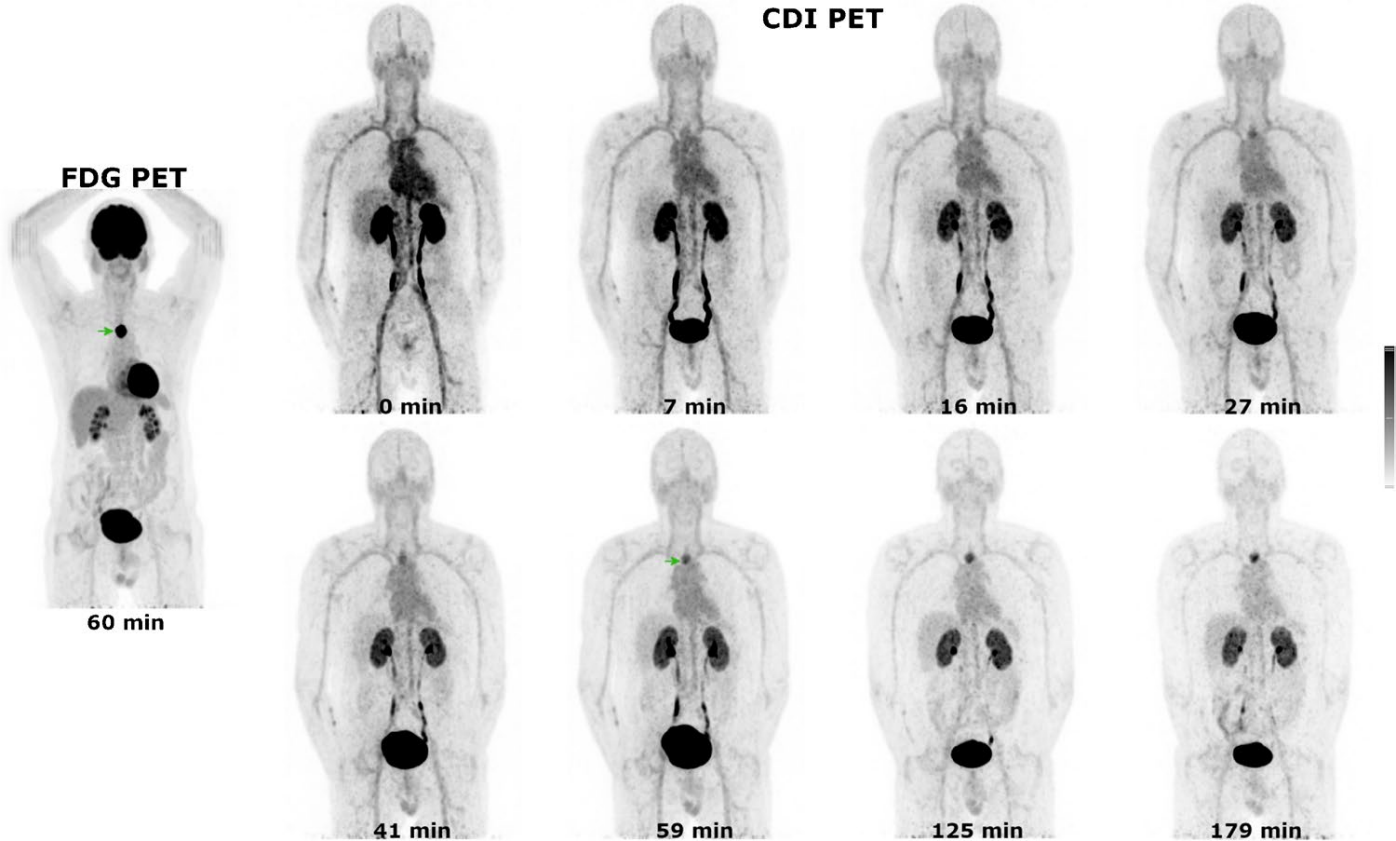
Maximum intensity projection images of FDG PET and eight sequential CDI PET scans for participant 1 (11 bed positions). All images are scaled from SUV 0 to 7. The tumour is arrowed on the FDG PET and CDI PET performed at 59 min post CDI injection

Serial PET imaging demonstrates rapid distribution in the blood pool, renal uptake and excretion with low levels of physiologic uptake in the remaining organs. Representative images of one participant are shown in Fig. 2 (images of the remaining participants are in Supplemental Data).

### Tumour uptake

**Fig. 3:**
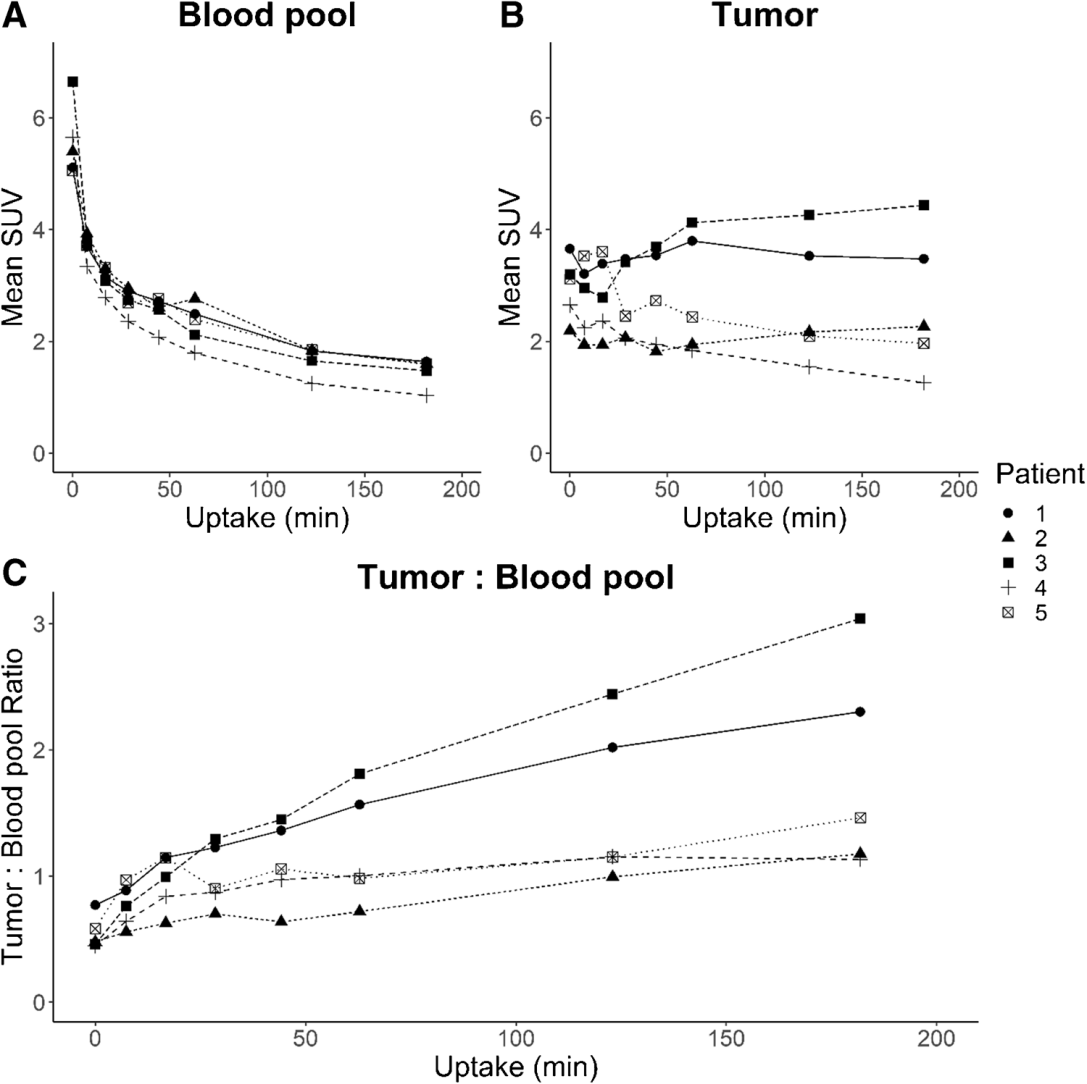
Mean SUV of the blood pool (**A**) and tumour (**B**) and tumour to blood pool ratio (**C**)

Tumour uptake is variable depending on tumour histology. High uptake is seen in squamous cell carcinoma of the oesophagus (SUVmax 5.7) and metastatic cutaneous squamous cell carcinoma (SUVmax 6.5), moderate uptake in metastatic colorectal carcinoma (SUVmax 4.4) and lower uptake in metastatic ovarian carcinoma (SUVmax 2.7) and breast carcinoma (SUVmax 2.5). In contrast to normal tissues and organs, tumour in 4 of the 5 patients demonstrated prolonged retention throughout the duration of imaging, with a commensurate increase in tumour to blood as blood pool activity progressively declined (Fig. 3).

### Radiation dosimetry

The average effective dose is 2.17E − 02 ± 4.61E − 03 mSv/MBq. Average organ doses are shown in Table 3. In all cases, the dose limiting organ was the urinary bladder wall (2.79E − 01 ± 1.11E − 01 mSv/MBq) followed by the kidneys (4.89E − 02 ± 3.55E − 03 mSv/MBq). Individual participant dosimetry is in the Supplemental Data.

**Table 3:** Average radiation dose for individual organs (*SD* standard deviation, *%COV* percentage coefficient of variation, *LLI* lower large intestine, *ULI* upper large intestine)

	Estimated radiation dose (mSv/MBq)		
Target organ	Average	SD	%COV
Adrenals	1.28E − 02	4.21E − 03	3.28E + 01
Brain	1.75E − 03	2.96E − 04	1.69E + 01
Breasts	7.03E − 03	3.52E − 03	5.01E + 01
Gallbladder wall	8.16E − 03	1.42E − 03	1.74E + 01
LLI wall	1.91E − 02	5.87E − 03	3.07E + 01
Small intestine	1.45E − 02	3.59E − 03	2.47E + 01
Stomach wall	1.00E − 02	3.76E − 03	3.76E + 01
ULI wall	1.23E − 02	4.88E − 03	3.96E + 01
Heart wall	1.02E − 02	1.95E − 03	1.90E + 01
Kidneys	4.89E − 02	3.55E − 03	7.26E + 00
Liver	1.61E − 02	3.54E − 03	2.19E + 01
Lungs	7.75E − 03	1.07E − 03	1.38E + 01
Muscle	8.96E − 03	2.17E − 03	2.42E + 01
Ovaries	1.20E − 02	3.00E − 03	2.51E + 01
Pancreas	1.52E − 02	5.12E − 03	3.38E + 01
Red marrow	8.89E − 03	1.33E − 03	1.50E + 01
Osteogenic cells	1.13E − 02	3.03E − 03	2.69E + 01
Skin	5.39E − 03	1.41E − 03	2.61E + 01
Spleen	1.59E − 02	3.63E − 03	2.28E + 01
Testes	1.67E − 02	3.26E − 03	1.95E + 01
Thymus	6.32E − 03	1.65E − 03	2.60E + 01
Thyroid	1.26E − 02	3.89E − 03	3.08E + 01
Urinary bladder wall	2.79E − 01	1.11E − 01	3.97E + 01
Uterus	2.63E − 02	1.17E − 03	4.43E + 00
Total Body	9.66E − 03	1.57E − 03	1.63E + 01
Effective dose	2.17E − 02	4.61E − 03	2.13E − 01

### Histological correlation

**Fig. 4:**
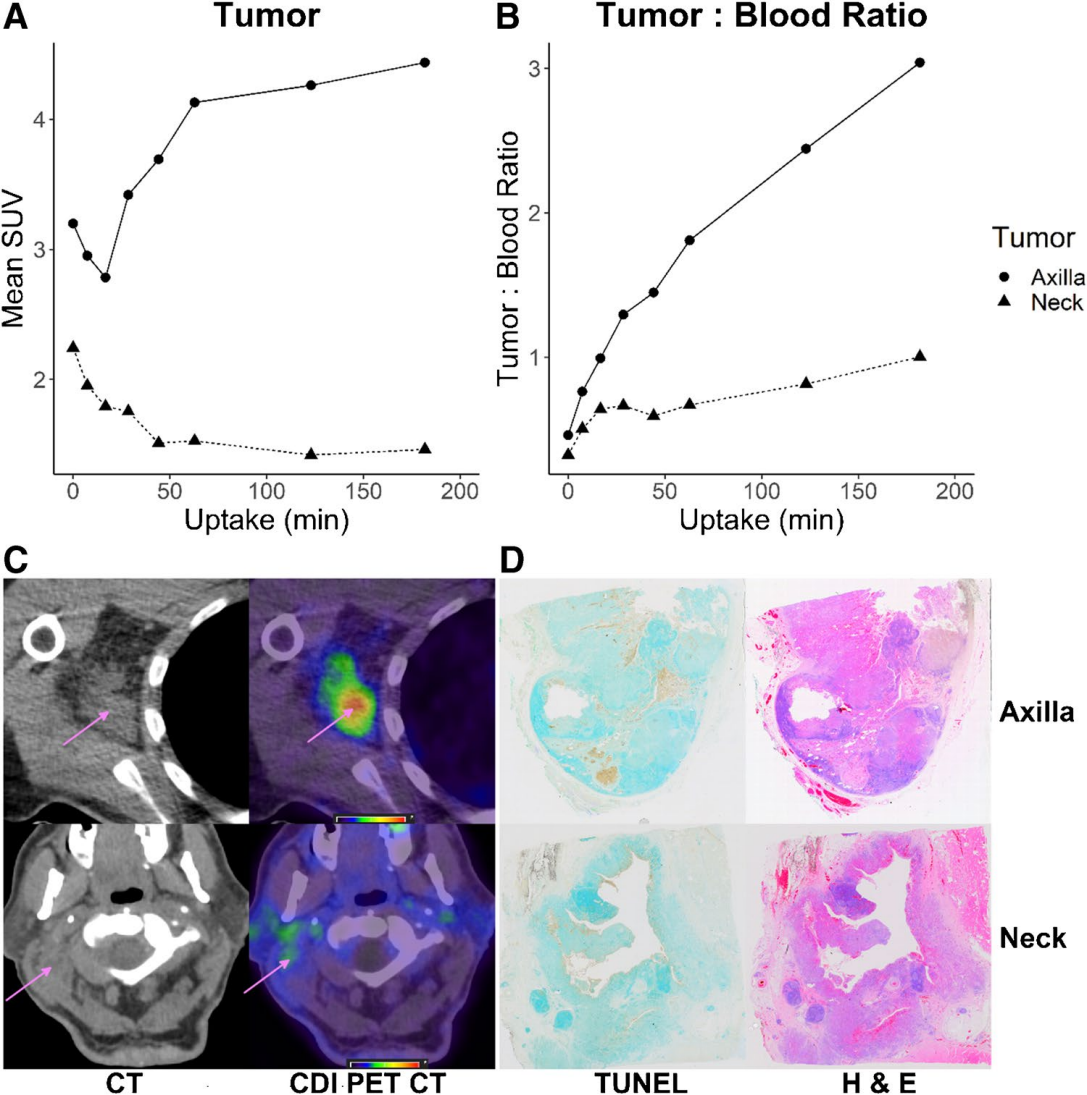
Mean SUV (**A**) and tumour to blood ratio (**B**) of the right cervical (neck) and right axillary squamous cell carcinoma lymph node metastases. Representative axial non contrast CT and fused CDI PET CT images (SUV 0–7) of the right axillary (top) and right cervical (bottom) squamous cell carcinoma lymph node metastases (**C**). Representative histological sections (not directly correlating to the PET CT slice) of the right axillary (top row) and right cervical (neck, bottom row) squamous cell carcinoma lymph node metastases stained with TUNEL (left, TUNEL-positive cells stain brown, arrowheads) and haematoxylin and eosin (right) (**D**)

Participant 3 presented with a nodal mass in the right axilla; biopsy of which demonstrated squamous cell carcinoma, thought to be metastatic from a previously excised cutaneous primary. During pre-operative assessment prior to resection of the right axillary nodal metastatic disease, CT and FDG PET CT demonstrated further nodal metastatic disease in the right upper neck. Given the anatomical location and clinical history (the participant previously had multiple cutaneous squamous cell carcinomas resected from the head, neck and trunk), the treating surgeon considered that this was likely to represent metastatic disease from a different cutaneous primary to that responsible for the right axillary nodal metastatic disease. The patient proceeded to right lateral neck dissection and right axillary dissection 1 day after the [^68^Ga]Ga-CDI PET study. Representative sections of the right axillary nodal metastases demonstrated many more TUNEL-positive cells than the right cervical nodal metastases. Corresponding haematoxylin and eosin–stained sections demonstrated that the TUNEL-positive cells corresponded to squamous cell carcinoma and adjacent normal lymphoid tissue did not demonstrate TUNEL staining. The number of TUNEL-positive cells correlated with the intensity of [^68^Ga]Ga-CDI uptake (Fig. 4).

## Discussion

This first-in-human study reports on a novel radiopharmaceutical, [^68^Ga]Ga-CDI, for imaging of cell death that is safe, well-tolerated, has dosimetry similar to clinical ^68^ Ga radiopharmaceuticals and demonstrates excellent biodistribution and imaging characteristics. [^68^ Ga]Ga-CDI overcomes most of the limitations encountered with previous attempts at molecular imaging of cell death and creates opportunities for personalised treatment based on near real-time imaging of treatment-induced cell death in cancer.

The safety, tolerability and absence of side effects and adverse events from [^68^Ga]Ga-CDI were an expected observation. Although [^68^Ga]Ga-CDI contains an arsenic atom, the active phenylarsenous targeting moiety of CDI, GSAO, has been previously assessed in a phase I study in patients with advanced solid tumours as a mitochondrial toxin. GSAO was infused 5 days per week for 2 weeks in every three and found to have a maximum tolerated dose of 22 mg/m^2^/day, which is almost 1000-fold greater than the administered mass in this study [19].

This study utilised dosimetry methodology employed for other ^68^Ga radiopharmaceuticals currently in clinical practice. Walker et al. reported the dosimetry of [^68^Ga]Ga-dotatate in 6 participants and found that the mean effective dose was 2.57E − 02 mSv/MBq and renal dose was 9.21E − 02 mSv/MBq [20]. A study comprising 4 participants found [^68^Ga]Ga-PSMA had a mean effective dose of 2.3E − 02 mSv/MBq, renal dose of 2.6E − 01 mSv/MBq and a urinary bladder dose of 1.3E − 01 mSv/MBq [21]. Another study of [^68^Ga]Ga-PSMA dosimetry which included 5 participants reported an effective dose of 2.37E − 02 mSv/MBq, renal dose of 1.21E − 01 mSv/MBq and urinary bladder dose of 1.64E − 01 mSv/MBq [22]. In summary, compared to both [^68^Ga]Ga-dotatate and [^68^Ga]-PSMA, [^68^Ga]Ga-CDI has a slightly lower effective dose and renal dose but a slightly higher urinary bladder dose and is therefore well-suited for routine and repeated human use.

Aside from the renal tract, which is the route of excretion, physiologic uptake of [^68^ Ga]Ga-CDI is low. In comparison, the activated caspase 3/7 agent, [^18^F]ICMT, has high levels of uptake in the hepatobiliary system and bowel as it undergoes both hepatobiliary and renal excretion [8]. Similarly, many annexin V radiopharmaceuticals demonstrate very high physiologic uptake in the kidneys (49.7% IA) and liver (13.1% IA) and prolonged retention [6]. The biodistribution characteristics of [^68^Ga]Ga-CDI translate to ideal imaging characteristics with minimal or no interference from physiologic uptake, whereas high physiologic uptake observed with other cell death imaging radiopharmaceuticals especially in the hepatobiliary system and bowel makes abdominal and pelvic imaging suboptimal. Furthermore, as CDI is labelled with ^68^ Ga, it enables short-interval serial PET for near real time, quantitative dynamic assessment of treatment-induced changes in tumour cell death.

Whilst acceptable dosimetry, good biodistribution and imaging characteristics are important, for a cell death imaging agent to be successful, it must have the sensitivity to quantitively detect tumour cell death. In vitro assessments of fluorophore conjugates of GSAO demonstrated rapid and specific uptake in dying/dead tumour cells. Half-maximal uptake in cultured tumour cells occurred in ~ 2 min and the specificity for dying/dead versus viable cells was at least 1000-fold [10]. In this study, [^68^ Ga]Ga-CDI detected constitutive tumour cell death with levels of [^68^ Ga]Ga-CDI uptake varying between different tumour types but also within tumours of the same histological subtype. The variations in intensity of uptake in different tumours is likely due to varying rates of constitutive tumour cell death. Tumour constitutive necrosis correlates with tumour aggressiveness [23]. Participants 2 and 5 both had metastatic disease which remained relatively stable for many months without active treatment, suggesting relatively indolent behaviour and hence likely low rates of constitutive tumour necrosis underlying the low level of [^68^Ga]Ga-CDI uptake in their tumour deposits. In addition, [^68^Ga]Ga-CDI tumour uptake correlated with histological tumour cell death assessed by TUNEL on operative specimens. Tumour uptake generally peaks at approximately at 60 min post injection and remains stable thereafter. However, tumour:blood pool ratios continue to rise beyond this due to progressive clearance of activity from the blood pool. Taken together and considering the half-life of ^68^Ga, it is likely that the optimal imaging time is between 1 and 2 h after injection.

The ability to detect constitutive tumour cell death demonstrates the sensitivity of [^68^Ga]Ga-CDI for imaging tumour cell death. In contrast, previous radiopharmaceuticals for imaging cell death did not reliably detect tumour cell death even after treatment. For example, the caspase ligand [^18^F]ICMT did not detect an increase in cell death in a proof-of-concept study. This was attributed to the low abundance and spatial and temporal heterogeneity of the target and that response may occur by mechanisms independent of caspase 3/7 activation [9]. Gammon et al. suggested that a long integration would be required to overcome these limitations [24]. [^68^Ga]Ga-CDI resolves these limitations. Firstly, hsp90 is highly abundant, constituting up to 1–2% of total cellular protein content and is often upregulated in malignancy [25], providing many potential binding sites in each dead and dying tumour cell. This makes hsp90 an ideal target for imaging cell death and it is likely that the high abundance of hsp90 underlies the ability of [^68^Ga]Ga-CDI to image even small numbers of dead and dying tumour cells such as occurs with constitutive tumour cell death. Furthermore, even in tumours with low rate of constitutive cell death (and hence low [^68^ Ga]Ga-CDI uptake prior to treatment), [^68^ Ga]Ga-CDI is still likely to be able to detect treatment response as even a small increase in tumour cell death following treatment should be detectable with [^68^Ga]Ga-CDI PET. Secondly, it is a spatially and temporally stable target, as once a cell is committed to death, the target is accessible and stable until the cellular debris is cleared by physiologic mechanisms, which may take several weeks. The optimal time for imaging tumour cell death with [^68^Ga]Ga-CDI after commencing treatment remains to be determined. Until now, effective methods for imaging tumour cell death in vivo have not been available to image the kinetics of tumour cell death following therapy and it is possible that this may vary between different treatment classes and modalities. Additional preclinical and clinical kinetic studies are warranted to further characterise this. However, the combination of preclinical studies with fluorophore-labelled GSAO demonstrating an increase in treatment induced tumour cell death 1 day following a single dose of chemotherapy, and the likely slow clearance of dead and dying cells from the tumour bed suggests that optimal imaging is likely to be within days to a few weeks after commencing treatment. Finally, in contrast to previous cell death imaging radiopharmaceuticals which have largely targeted aspects of apoptosis, [^68^Ga]Ga-CDI detects different forms of cell death and there is increasing evidence of the importance of non-apoptotic cell death in malignancy [26].

The development of optimal and widely accessible molecular imaging of tumour cell death creates many new opportunities to improve outcomes in oncology. Most obviously, cell death imaging has the potential to assess treatment response more accurately and rapidly. Although current imaging modalities such as CT, FDG PET and MRI have proven utility for assessing treatment response, in many circumstances they are suboptimal. For example, following radiotherapy treatment, related effects such as fibrosis and post radiotherapy inflammatory change make anatomic and molecular imaging inaccurate [27]. Accurate imaging assessment of treatment response in immuno-oncology is another major unmet need. In such circumstances, imaging assessment of treatment response may be inaccurate and often delayed until treatment-induced effects have subsided. Imaging of changes in tumour cell death following treatment provides the opportunity for much earlier and more accurate assessment of treatment response in such situations.

Imaging-guided response–adapted individualised treatment provides the opportunity to improve outcomes in oncology but is predicated by imaging that provides timely and sufficiently accurate response assessment. With the possible exception of FDG PET in Hodgkin’s and non-Hodgkin’s lymphoma, current imaging modalities are inadequate. For example, in breast carcinoma being treated with neoadjuvant systemic therapy, MRI and FDG PET are moderately accurate for prediction of pathological response; however, neither are sufficiently accurate to avoid unnecessary surgery in patients who have a complete pathological response following neoadjuvant therapy [28]. Similar challenges exist in oesophageal and rectal carcinoma with neoadjuvant therapy [29]. Even when imaging is combined with clinical and histological assessment, it remains inadequate to determine if patients should proceed to surgery or may be safely managed with a “watch and wait” approach. Beyond clinical care, cell death imaging provides significant opportunities to improve the conduct of clinical trials by imaging a specific and direct biomarker to identify active agents or combinations. Furthermore, cell death imaging of healthy organs and tissues will provide direct assessment of toxicity and may enable rational customised dose escalation strategies.

## Conclusions

[^68^Ga]Ga-CDI is a novel radiopharmaceutical for imaging of cell death. It is safe, has radiation dosimetry similar to clinical ^68^Ga radiopharmaceuticals and demonstrates optimal biodistribution and imaging characteristics. It is able to detect constitutive tumour cell death and uptake correlates with histological cell death. It is now entering a proof-of-concept trial and has the potential to improve treatment response assessment and enable cell death imaging–adapted personalised treatment approaches.

## Supplementary Information

Below is the link to the electronic supplementary material.

Supplementary file1 (DOCX 8486 KB)

## Data Availability

Anonymised source data from this study is available with investigator support, after approval of a proposal and with a signed data access agreement.

## Abbreviations

CDI: Cell death indicator
CT: X-ray computed tomography
DICOM: Digital imaging and communications in medicine
DOTA: 1,4,7,10-Tetraazacyclododecane-1,4,7,10-tetraacetic acid
DTPA: Diethylene triamine pentaacetic acid
ECG: Electrocardiograph
FDG: 2-Fluoro-2-deoxyglucose
GSAO: 4-(N-(S-glutathionylacetyl)amino)phenylarsonous acid
HPLC: High performance liquid chromatography
hsp90: Heat shock 90 proteins
LC-MS: Liquid chromatography – mass spectroscopy
MRI: Magnetic resonance imaging
NODAGA: 2-(4,7-bis(carboxymethyl)-1,4,7-triazonan-1-yl)pentanedioic acid
PET: Positron emission tomography
RECIST: Response evaluation criteria in solid tumours
TLC: Thin layer chromatography
TUNEL: Terminal deoxynucleotidyl transferase dUTP nick end labelling
VOI: Volume of interest
