# Supplementary material for: A first-in-human study of [68Ga]Ga-CDI: a positron emitting radiopharmaceutical for imaging tumour cell death
Source: Eur J Nucl Med Mol Imaging. 2022 Jul 2;49(12):4037–47. doi: 10.1007/s00259-022-05880-z (PMC9525422; doi:10.1007/s00259-022-05880-z)
Supplement: Supplementary file 1 — Supplementary file1 (DOCX 8486 KB) [file 259_2022_5880_MOESM1_ESM.docx]

# Supplemental data to “A first-in-human study of [^68^Ga]Ga-CDI: a novel positron emitting radiopharmaceutical for imaging tumour cell death.”


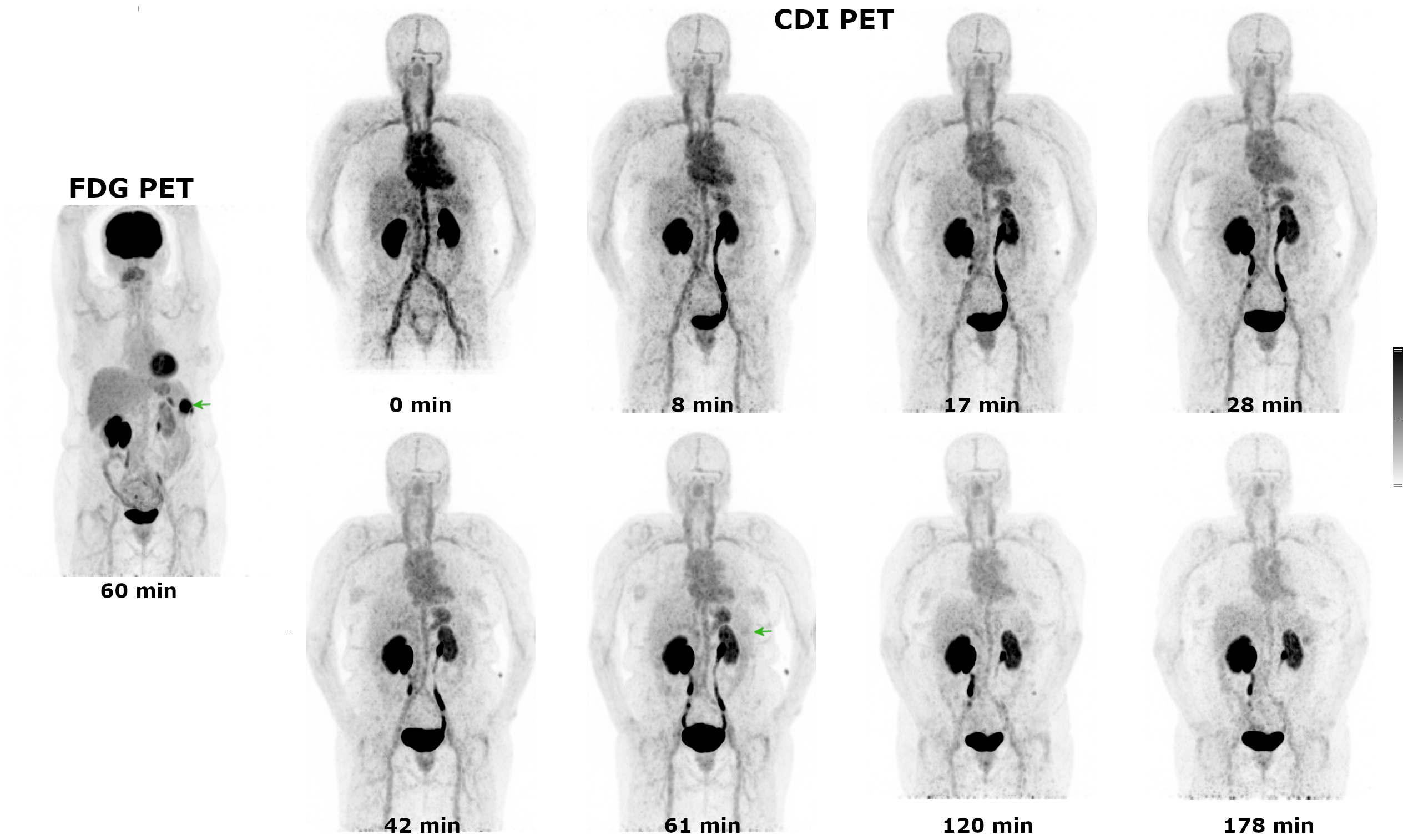


Figure 1: Maximum intensity projection images of FDG PET and eight sequential CDI PET scans for participant 2 (11 bed positions). All images are scaled from SUV 0 to 7. A representative tumour site is arrowed on the FDG PET and CDI PET performed at 61 minutes post CDI injection.


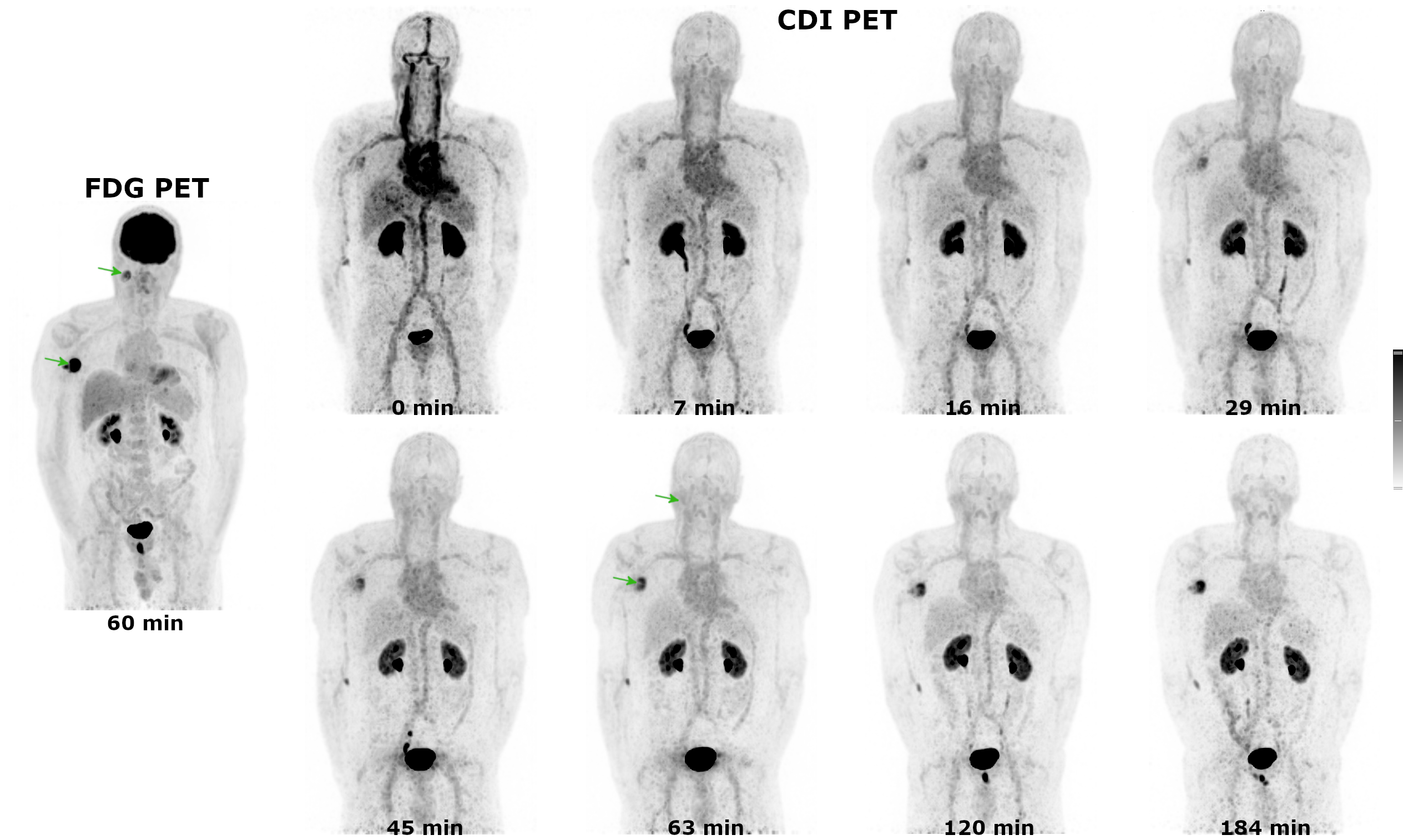


Figure 2: Maximum intensity projection images of FDG PET and eight sequential CDI PET scans for participant 3 (11 bed positions). All images are scaled from SUV 0 to 7. Two tumour sites arrowed on the FDG PET and CDI PET performed at 63 minutes post CDI injection.


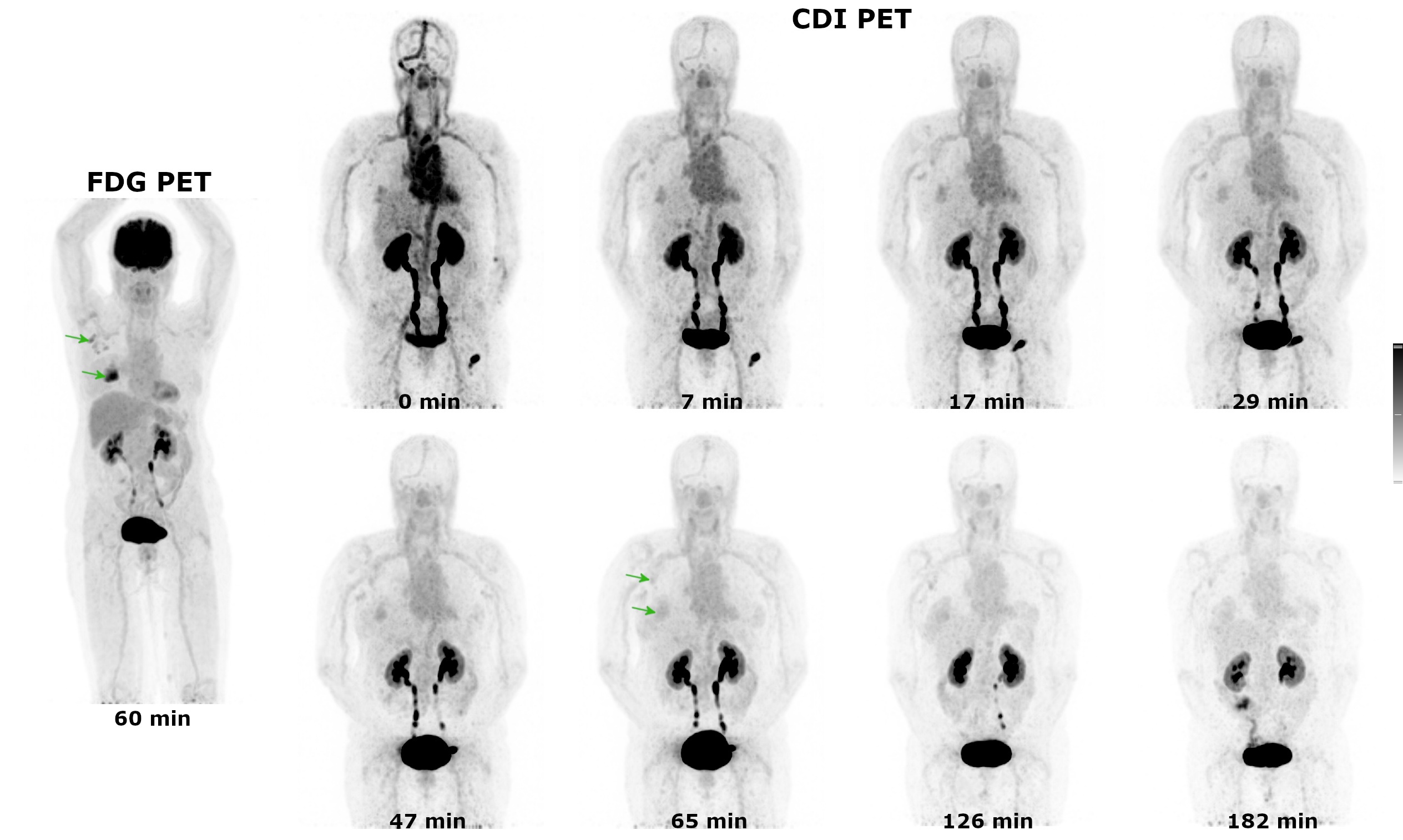


Figure 3: Maximum intensity projection images of FDG PET and eight sequential CDI PET scans for participant 4 (10 bed positions). All images are scaled from SUV 0 to 7. Two representative tumours site are arrowed on the FDG PET and CDI PET performed at 65 minutes post CDI injection.


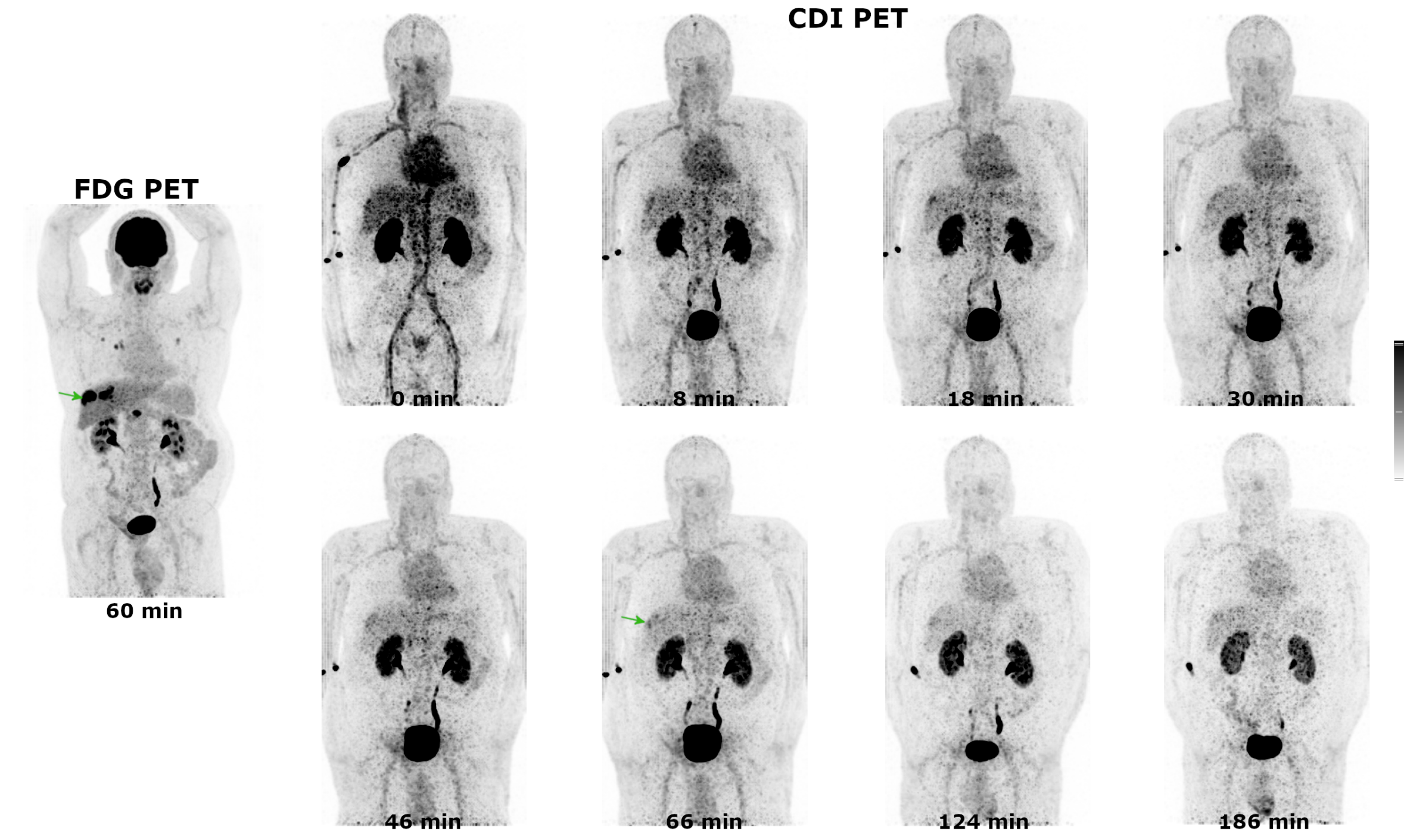


Figure 4: Maximum intensity projection images of FDG PET and eight sequential CDI PET scans for participant 5 (12 bed positions). All images are scaled from SUV 0 to 7. A representative tumour site is arrowed on the FDG PET and CDI PET performed at 66 minutes post CDI injection.

|  | **Estimated radiation dose (mSv/MBq)** | | | | |
| --- | --- | --- | --- | --- | --- |
| **Target Organ** | **Alpha** | **Beta** | **Photon** | **Total** | **ED Cont.** |
| Adrenals | 0.00E+00 | 1.02E-02 | 4.25E-03 | 1.44E-02 | 8.86E-05 |
| Brain | 0.00E+00 | 6.97E-04 | 1.00E-03 | 1.70E-03 | 1.70E-05 |
| Breasts | 0.00E+00 | 2.34E-03 | 1.82E-03 | 4.16E-03 | 3.33E-04 |
| Gallbladder Wall | 0.00E+00 | 3.10E-03 | 4.56E-03 | 7.66E-03 | 4.71E-05 |
| LLI Wall | 0.00E+00 | 2.01E-02 | 7.31E-03 | 2.74E-02 | 1.64E-03 |
| Small Intestine | 0.00E+00 | 1.17E-02 | 5.30E-03 | 1.70E-02 | 1.05E-04 |
| Stomach Wall | 0.00E+00 | 5.16E-03 | 3.62E-03 | 8.78E-03 | 1.05E-03 |
| ULI Wall | 0.00E+00 | 1.07E-02 | 5.28E-03 | 1.60E-02 | 9.60E-04 |
| Heart Wall | 0.00E+00 | 6.11E-03 | 3.32E-03 | 9.43E-03 | 5.80E-05 |
| Kidneys | 0.00E+00 | 4.32E-02 | 7.12E-03 | 5.04E-02 | 3.10E-04 |
| Liver | 0.00E+00 | 1.28E-02 | 4.79E-03 | 1.76E-02 | 7.04E-04 |
| Lungs | 0.00E+00 | 5.04E-03 | 2.66E-03 | 7.70E-03 | 9.24E-04 |
| Muscle | 0.00E+00 | 5.84E-03 | 3.30E-03 | 9.14E-03 | 5.62E-05 |
| Ovaries | NA | NA | NA | NA | NA |
| Pancreas | 0.00E+00 | 8.84E-03 | 4.47E-03 | 1.33E-02 | 8.18E-05 |
| Red Marrow | 0.00E+00 | 5.40E-03 | 3.48E-03 | 8.88E-03 | 1.07E-03 |
| Osteogenic Cells | 0.00E+00 | 6.01E-03 | 3.19E-03 | 9.19E-03 | 9.19E-05 |
| Skin | 0.00E+00 | 2.34E-03 | 1.89E-03 | 4.23E-03 | 4.23E-05 |
| Spleen | 0.00E+00 | 1.33E-02 | 4.39E-03 | 1.77E-02 | 1.09E-04 |
| Testes | 0.00E+00 | 1.51E-02 | 4.97E-03 | 2.01E-02 | 1.61E-03 |
| Thymus | 0.00E+00 | 2.34E-03 | 2.66E-03 | 5.00E-03 | 3.08E-05 |
| Thyroid | 0.00E+00 | 1.12E-02 | 2.81E-03 | 1.40E-02 | 5.60E-04 |
| Urinary Bladder Wall | 0.00E+00 | 2.28E-01 | 3.33E-02 | 2.62E-01 | 1.05E-02 |
| Uterus | NA | NA | NA | NA | NA |
| Total Body | 0.00E+00 | 5.66E-03 | 3.22E-03 | 8.89E-03 | 1.33E-03 |
| **Effective Dose** |  |  |  |  | **2.17E-02** |

Table 1: Dosimetry for participant 1 (ED Cont = Effective dose contribution; ULI = upper large intestine, LLI = lower large intestine).

|  | **Estimated radiation dose (mSv/MBq)** | | | | |
| --- | --- | --- | --- | --- | --- |
| **Target Organ** | **Alpha** | **Beta** | **Photon** | **Total** | **ED Cont.** |
| Adrenals | 0.00E+00 | 9.53E-03 | 5.24E-03 | 1.48E-02 | 9.11E-05 |
| Brain | 0.00E+00 | 6.40E-04 | 1.46E-03 | 2.10E-03 | 2.10E-05 |
| Breasts | 0.00E+00 | 9.48E-03 | 2.76E-03 | 1.22E-02 | 9.76E-04 |
| Gallbladder Wall | 0.00E+00 | 4.69E-03 | 5.27E-03 | 9.96E-03 | 6.13E-05 |
| LLI Wall | 0.00E+00 | 1.37E-02 | 7.16E-03 | 2.08E-02 | 1.25E-03 |
| Small Intestine | 0.00E+00 | 1.27E-02 | 5.57E-03 | 1.83E-02 | 1.13E-04 |
| Stomach Wall | 0.00E+00 | 1.15E-02 | 4.97E-03 | 1.65E-02 | 1.98E-03 |
| ULI Wall | 0.00E+00 | 1.19E-02 | 6.02E-03 | 1.79E-02 | 1.07E-03 |
| Heart Wall | 0.00E+00 | 8.27E-03 | 4.29E-03 | 1.26E-02 | 7.75E-05 |
| Kidneys | 0.00E+00 | 4.21E-02 | 7.59E-03 | 4.97E-02 | 3.06E-04 |
| Liver | 0.00E+00 | 1.45E-02 | 5.51E-03 | 2.00E-02 | 8.00E-04 |
| Lungs | 0.00E+00 | 5.73E-03 | 3.54E-03 | 9.27E-03 | 1.11E-03 |
| Muscle | 0.00E+00 | 6.08E-03 | 3.83E-03 | 9.91E-03 | 6.10E-05 |
| Ovaries | 0.00E+00 | 4.69E-03 | 6.95E-03 | 1.16E-02 | 9.28E-04 |
| Pancreas | 0.00E+00 | 1.62E-02 | 5.90E-03 | 2.21E-02 | 1.36E-04 |
| Red Marrow | 0.00E+00 | 6.61E-03 | 4.24E-03 | 1.08E-02 | 1.30E-03 |
| Osteogenic Cells | 0.00E+00 | 1.14E-02 | 4.14E-03 | 1.55E-02 | 1.55E-04 |
| Skin | 0.00E+00 | 4.69E-03 | 2.32E-03 | 7.01E-03 | 7.01E-05 |
| Spleen | 0.00E+00 | 1.48E-02 | 5.34E-03 | 2.02E-02 | 1.24E-04 |
| Testes | NA | NA | NA | NA | NA |
| Thymus | 0.00E+00 | 4.69E-03 | 3.66E-03 | 8.36E-03 | 5.14E-05 |
| Thyroid | 0.00E+00 | 1.19E-02 | 3.24E-03 | 1.52E-02 | 6.08E-04 |
| Urinary Bladder Wall | 0.00E+00 | 1.84E-01 | 3.21E-02 | 2.16E-01 | 8.64E-03 |
| Uterus | 0.00E+00 | 4.69E-03 | 8.98E-03 | 1.37E-02 | 8.43E-05 |
| Total Body | 0.00E+00 | 7.76E-03 | 3.84E-03 | 1.16E-02 | 1.74E-03 |
| **Effective Dose** |  |  |  |  | **2.18E-02** |

Table 2: Dosimetry for participant 2 (ED Cont = Effective dose contribution; ULI = upper large intestine, LLI = lower large intestine).

|  | **Estimated radiation dose (mSv/MBq)** | | | | |
| --- | --- | --- | --- | --- | --- |
| **Target Organ** | **Alpha** | **Beta** | **Photon** | **Total** | **ED Cont.** |
| Adrenals | 0.00E+00 | 5.97E-03 | 3.71E-03 | 9.68E-03 | 5.96E-05 |
| Brain | 0.00E+00 | 6.13E-04 | 8.65E-04 | 1.48E-03 | 1.48E-05 |
| Breasts | 0.00E+00 | 2.02E-03 | 1.59E-03 | 3.61E-03 | 2.89E-04 |
| Gallbladder Wall | 0.00E+00 | 2.02E-03 | 4.07E-03 | 6.09E-03 | 3.75E-05 |
| LLI Wall | 0.00E+00 | 1.44E-02 | 5.96E-03 | 2.03E-02 | 1.22E-03 |
| Small Intestine | 0.00E+00 | 8.73E-03 | 4.29E-03 | 1.30E-02 | 8.00E-05 |
| Stomach Wall | 0.00E+00 | 4.31E-03 | 3.12E-03 | 7.43E-03 | 8.92E-04 |
| ULI Wall | 0.00E+00 | 2.02E-03 | 4.00E-03 | 6.02E-03 | 3.61E-04 |
| Heart Wall | 0.00E+00 | 4.65E-03 | 2.82E-03 | 7.48E-03 | 4.60E-05 |
| Kidneys | 0.00E+00 | 4.45E-02 | 6.83E-03 | 5.13E-02 | 3.16E-04 |
| Liver | 0.00E+00 | 1.20E-02 | 4.36E-03 | 1.64E-02 | 6.56E-04 |
| Lungs | 0.00E+00 | 5.16E-03 | 2.37E-03 | 7.53E-03 | 9.04E-04 |
| Muscle | 0.00E+00 | 5.15E-03 | 2.83E-03 | 7.98E-03 | 4.91E-05 |
| Ovaries | NA | NA | NA | NA | NA |
| Pancreas | 0.00E+00 | 1.13E-02 | 4.18E-03 | 1.54E-02 | 9.48E-05 |
| Red Marrow | 0.00E+00 | 4.29E-03 | 2.96E-03 | 7.25E-03 | 8.70E-04 |
| Osteogenic Cells | 0.00E+00 | 4.95E-03 | 2.74E-03 | 7.69E-03 | 7.69E-05 |
| Skin | 0.00E+00 | 2.02E-03 | 1.63E-03 | 3.66E-03 | 3.66E-05 |
| Spleen | 0.00E+00 | 1.09E-02 | 3.83E-03 | 1.47E-02 | 9.05E-05 |
| Testes | 0.00E+00 | 1.22E-02 | 4.18E-03 | 1.64E-02 | 1.31E-03 |
| Thymus | 0.00E+00 | 2.02E-03 | 2.30E-03 | 4.32E-03 | 2.66E-05 |
| Thyroid | 0.00E+00 | 8.46E-03 | 2.38E-03 | 1.08E-02 | 4.32E-04 |
| Urinary Bladder Wall | 0.00E+00 | 1.91E-01 | 2.79E-02 | 2.19E-01 | 8.76E-03 |
| Uterus | NA | NA | NA | NA | NA |
| Total Body | 0.00E+00 | 4.94E-03 | 2.77E-03 | 7.71E-03 | 1.16E-03 |
| **Effective Dose** |  |  |  |  | **1.78E-02** |

Table 3: Dosimetry for participant 3 (ED Cont = Effective dose contribution; ULI = upper large intestine, LLI = lower large intestine).

|  | **Estimated radiation dose (mSv/MBq)** | | | | |
| --- | --- | --- | --- | --- | --- |
| **Target Organ** | **Alpha** | **Beta** | **Photon** | **Total** | **ED Cont.** |
| Adrenals | 0.00E+00 | 1.30E-02 | 4.86E-03 | 1.79E-02 | 1.10E-04 |
| Brain | 0.00E+00 | 7.79E-04 | 1.23E-03 | 2.01E-03 | 2.01E-05 |
| Breasts | 0.00E+00 | 6.43E-03 | 2.25E-03 | 8.68E-03 | 6.94E-04 |
| Gallbladder Wall | 0.00E+00 | 4.09E-03 | 4.46E-03 | 8.56E-03 | 5.27E-05 |
| LLI Wall | 0.00E+00 | 3.53E-03 | 8.79E-03 | 1.23E-02 | 7.38E-04 |
| Small Intestine | 0.00E+00 | 3.53E-03 | 5.67E-03 | 9.20E-03 | 5.66E-05 |
| Stomach Wall | 0.00E+00 | 3.53E-03 | 4.01E-03 | 7.54E-03 | 9.05E-04 |
| ULI Wall | 0.00E+00 | 3.53E-03 | 5.50E-03 | 9.03E-03 | 5.42E-04 |
| Heart Wall | 0.00E+00 | 7.57E-03 | 3.84E-03 | 1.14E-02 | 7.02E-05 |
| Kidneys | 0.00E+00 | 4.30E-02 | 7.33E-03 | 5.03E-02 | 3.10E-04 |
| Liver | 0.00E+00 | 1.16E-02 | 4.71E-03 | 1.63E-02 | 6.52E-04 |
| Lungs | 0.00E+00 | 4.82E-03 | 3.15E-03 | 7.97E-03 | 9.56E-04 |
| Muscle | 0.00E+00 | 7.68E-03 | 4.07E-03 | 1.18E-02 | 7.26E-05 |
| Ovaries | 0.00E+00 | 3.53E-03 | 8.79E-03 | 1.23E-02 | 9.84E-04 |
| Pancreas | 0.00E+00 | 1.18E-02 | 5.04E-03 | 1.69E-02 | 1.04E-04 |
| Red Marrow | 0.00E+00 | 5.25E-03 | 4.13E-03 | 9.38E-03 | 1.13E-03 |
| Osteogenic Cells | 0.00E+00 | 8.81E-03 | 3.80E-03 | 1.26E-02 | 1.26E-04 |
| Skin | 0.00E+00 | 3.53E-03 | 2.26E-03 | 5.79E-03 | 5.79E-05 |
| Spleen | 0.00E+00 | 1.18E-02 | 4.70E-03 | 1.65E-02 | 1.02E-04 |
| Testes | NA | NA | NA | NA | NA |
| Thymus | 0.00E+00 | 3.53E-03 | 3.23E-03 | 6.76E-03 | 4.16E-05 |
| Thyroid | 0.00E+00 | 1.34E-02 | 3.00E-03 | 1.64E-02 | 6.56E-04 |
| Urinary Bladder Wall | 0.00E+00 | 4.06E-01 | 6.85E-02 | 4.74E-01 | 1.90E-02 |
| Uterus | 0.00E+00 | 2.38E-02 | 1.51E-02 | 3.89E-02 | 2.39E-04 |
| Total Body | 0.00E+00 | 6.96E-03 | 3.92E-03 | 1.09E-02 | 1.64E-03 |
| **Effective Dose** |  |  |  |  | **2.92E-02** |

Table 4: Dosimetry for participant 4 (ED Cont = Effective dose contribution; ULI = upper large intestine, LLI = lower large intestine).

|  | **Estimated radiation dose (mSv/MBq)** | | | | |
| --- | --- | --- | --- | --- | --- |
| **Target Organ** | **Alpha** | **Beta** | **Photon** | **Total** | **ED Cont.** |
| Adrenals | 0.00E+00 | 3.73E-03 | 3.71E-03 | 7.44E-03 | 4.58E-05 |
| Brain | 0.00E+00 | 2.83E-04 | 1.18E-03 | 1.46E-03 | 1.46E-05 |
| Breasts | 0.00E+00 | 4.26E-03 | 2.26E-03 | 6.52E-03 | 5.22E-04 |
| Gallbladder Wall | 0.00E+00 | 4.60E-03 | 3.93E-03 | 8.53E-03 | 5.25E-05 |
| LLI Wall | 0.00E+00 | 8.66E-03 | 6.19E-03 | 1.48E-02 | 8.88E-04 |
| Small Intestine | 0.00E+00 | 1.01E-02 | 5.13E-03 | 1.52E-02 | 9.35E-05 |
| Stomach Wall | 0.00E+00 | 6.23E-03 | 3.53E-03 | 9.76E-03 | 1.17E-03 |
| ULI Wall | 0.00E+00 | 7.83E-03 | 4.73E-03 | 1.26E-02 | 7.56E-04 |
| Heart Wall | 0.00E+00 | 6.79E-03 | 3.47E-03 | 1.03E-02 | 6.34E-05 |
| Kidneys | 0.00E+00 | 3.64E-02 | 6.24E-03 | 4.26E-02 | 2.62E-04 |
| Liver | 0.00E+00 | 6.86E-03 | 3.53E-03 | 1.04E-02 | 4.16E-04 |
| Lungs | 0.00E+00 | 3.67E-03 | 2.60E-03 | 6.27E-03 | 7.52E-04 |
| Muscle | 0.00E+00 | 2.73E-03 | 3.27E-03 | 5.99E-03 | 3.69E-05 |
| Ovaries | NA | NA | NA | NA | NA |
| Pancreas | 0.00E+00 | 4.27E-03 | 3.83E-03 | 8.09E-03 | 4.98E-05 |
| Red Marrow | 0.00E+00 | 4.64E-03 | 3.52E-03 | 8.16E-03 | 9.79E-04 |
| Osteogenic Cells | 0.00E+00 | 7.67E-03 | 3.64E-03 | 1.13E-02 | 1.13E-04 |
| Skin | 0.00E+00 | 4.26E-03 | 2.02E-03 | 6.28E-03 | 6.28E-05 |
| Spleen | 0.00E+00 | 6.88E-03 | 3.59E-03 | 1.05E-02 | 6.46E-05 |
| Testes | 0.00E+00 | 9.15E-03 | 4.42E-03 | 1.36E-02 | 1.09E-03 |
| Thymus | 0.00E+00 | 4.26E-03 | 2.92E-03 | 7.18E-03 | 4.42E-05 |
| Thyroid | 0.00E+00 | 4.13E-03 | 2.63E-03 | 6.76E-03 | 2.70E-04 |
| Urinary Bladder Wall | 0.00E+00 | 1.94E-01 | 2.86E-02 | 2.23E-01 | 8.92E-03 |
| Uterus | NA | NA | NA | NA | NA |
| Total Body | 0.00E+00 | 6.00E-03 | 3.21E-03 | 9.21E-03 | 1.38E-03 |
| **Effective Dose** |  |  |  |  | **1.80E-02** |

Table 5: Dosimetry for participant 5 (ED Cont = Effective dose contribution; ULI = upper large intestine, LLI = lower large intestine).
